# The General Medical Council

**Published:** 1895-12-07

**Authors:** 


### THE GENERAL MEDICAL COUNCIL.

The fifty-ninth session of the General Medical Council was opened on November 26th with the usual address from the president, Sir Richard Quain, introductory to the several matters of business to be brought forward; and it was closed on Monday December 2nd. The meetings were held in the library of the Royal College of Physicians, kindly lent for the purpose, the large room in the Council's house being in process of alteration.

The penal cases of the session were few in number, and not important in character. At the May meeting two practitioners, named respectively Richards and McCarthy, were found to have committed the offence which is technically known as "covering," that is to say, they had each enabled an unqualified person to carry on medical practice. The cases, however, were not of the worst kind, and the Council deferred decision upon them for six months in order to allow the defendants to abandon the practices complained of, and to produce evidence of having done so. This evidence having been produced and accepted as sufficient, the Council decided to take no further action in the matter, and not to remove Mr. Richards and Mr. McCarthy from the register. A Mr. Theobald, whose name was erased during the May session, applied for a re-hearing of his case, but, in the opinion of the Council, failed to show any cause why his application should be granted.

The most important business before the Council had reference to the position and claims of the Apothecaries' Hall of Ireland. That body was recognised, by the Medical Act of 1858, as having power to confer a medical qualification; but its position was materially affected by the Act of 1886, which provided that no qualification should be registrable unless it was valid for all the three branches of medicine, surgery, and midwifery. A body empowered to issue licences in one or two of these branches only was permitted to complete itself, so to speak, by combining with one capable of supplying its deficiencies; or, if unable to do this, was permitted to apply to the Medical Council for the appointment of additional examiners. Among the licensing bodies affected by this portion of the Act, two, the Society of Apothecaries of London and the Apothecaries' Hall of Ireland, were able to give a qualification in medicine and in midwifery, but were unable to give a qualification in surgery, so that their examinations, taken alone, did not entitle those who passed them to register. The Society of Apothecaries of London was not able to combine with any other licensing body for the supply of its deficiency, and hence examiners in surgery were granted to it by the Medical Council, so that it was placed in a position of independence as a complete qualifying body. The Apothecaries' Hall of Ireland combined with the Royal College of Surgeons of Ireland, and formed with that college a conjoint board having full powers and privileges. But one of the functions of the Medical Council is to inspect the examinations held by the various qualifying bodies, and to ascertain whether they are sufficient; the Council having the further duty, if any examination is found to be insufficient, of reporting it to the Privy Council, which then has power to suspend or withdraw the privilege of registration. The examination of the conjoint board formed by the Irish Hall and College has been unfavourably reported upon by the inspector and visitors deputed by the Council; and at last, after having been four times specially visited, was reported to the Privy Council in the manner required by law. The College of Surgeons, in consequence of the action of the Council, gave notice to withdraw from the combination, so that the board which they assisted to form has now ceased to exist, except with reference to students who had entered for examination prior to last July; and it remains to be seen whether the Privy Council will allow the board to continue its work until these students have presented themselves. In the meanwhile, the Irish Hall stands alone; and it made an application to the Council for the appointment of surgical examiners, who would place it upon the same footing as the Society of Apothecaries of London. After prolonged discussion, the Council decided to refuse the application, so that the Irish Hall will now cease to be a qualifying body. It has a right of appeal to the Privy Council, but there is not much probability that such an appeal will be successful. The occasion is the first on which the Medical Council has exercised the important disciplinary powers conferred upon it by the Medical Acts, and its deliberate decision on an essentially medical question is not likely to be disregarded by a non-medical authority.

The last two or three days of the session were devoted to the consideration of two highly important reports, and to the passing of recommendations based upon them. One of these reports was prepared by the Examinations Committee of the Council, upon the whole of the final examinations of the several licensing bodies; and the other by the Public Health Committee, upon the examinations for degrees or diplomas in Public Health or State Medicine. With regard to these, it rests with the Council alone, independently of the Privy Council, to determine whether or not they shall be registrable; and hence the Council is able to lay down stringent conditions with regard both to preliminary study and to actual examination. The principle governing these conditions is to secure that every diploma in Public Health shall imply the possession not merely of pass knowledge, but of a distinctively high proficiency in each and all of the various branches of the subject. Such diplomas are not required by ordinary practitioners, but only by those who seek to obtain appointments as medical officers of health to large urban or country districts. The recommendations based upon the reports of the final, or qualifying examinations for practice, were referred back to the Examinations Committee in order to be codified and put into proper shape for transmission to the licensing bodies concerned.

